# From the Front Lines of COVID-19 at HSS: An Oral History

**DOI:** 10.1007/s11420-020-09816-x

**Published:** 2020-11-05

**Authors:** C. Ronald MacKenzie, Joy Jacobson

**Affiliations:** 1 Division of Rheumatology, Department of Medicine, Hospital for Special Surgery, 535 East 70th Street, New York, NY 10021 USA; 2 Education Institute, Hospital for Special Surgery, 535 East 70th Street, New York, NY 10021 USA

**Keywords:** COVID-19, pandemic response, infectious disease, oral history

## Introduction

In mid-March 2020, at the start of New York City’s battle against COVID-19, the disease caused by the deadly severe acute respiratory syndrome coronavirus 2 (SARS-CoV-2), leaders at Hospital for Special Surgery (HSS) realized that in order to protect both patients and staff, the hospital would have to stop performing all nonessential surgery. Within 2 weeks, as the city’s facilities ran out of room to care for the crush of COVID-19 patients, the hospital’s leadership decided that HSS would transform itself into a hospital capable of treating COVID-19 patients.

On April 10, during one of the deadliest weeks of the pandemic in New York City, HSS president and CEO Louis Shapiro discussed “fighting the fight so we can win the war” and what “new normal” might become after the pandemic. “It would be a crime to let this crisis go to waste by not taking advantage of what we’ve learned so that we can be better on the other side,” he said. “We want everyone who can to take advantage of what we’ve learned.”

**Fig. 1:**
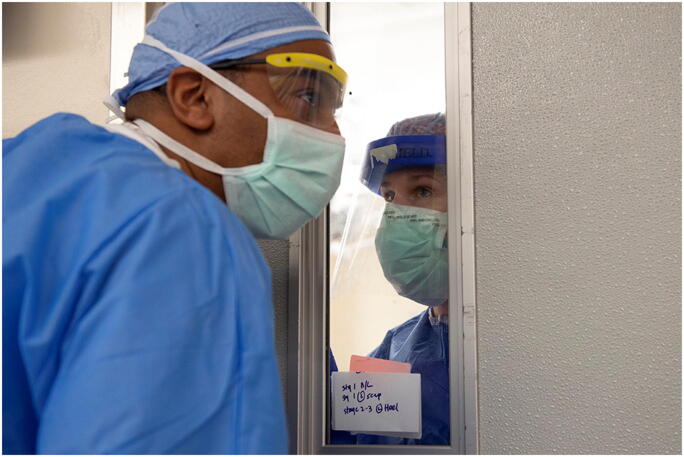
Mose Holmes, registrar, and McKenna Gibson, RN, at HSS, on April 14, 2020. Photo by Clay Woodland Englehart.

When asked what he’d been surprised by, Mr. Shapiro answered: “When you think you couldn’t be inspired by people anymore, and every day you see people coming together even more—I’ve never seen anything like it. There is nothing quite like being in a crisis of epic proportion and at the very same time being inspired by what people are doing” (Fig. 1).

Here we present first-person reflections on the battle against COVID-19 at HSS. Distilled from interviews conducted during the surge period of the crisis in New York City, March through May 2020, the oral history and letters from the front lines reveal the trajectory of the hospital’s transformation.—*C*. *Ronald MacKenzie*, *MD*, *guest editor*; *Joy Jacobson*, *managing editor*

## March 31, 2020

“Coronavirus may kill 100,000 to 240,000 in U.S. despite actions, officials say.”

*The New York Times*, March 31, 2020.

### Bryan Kelly, MD, MBA, HSS surgeon in chief and medical director

It’s March 31, I think. Tuesday. The days have started to blur into one another. Every day has been a whirlwind in terms of trying to figure out our best way to respond to this crisis. Two weeks ago we made what seemed like a monumental decision—to stop all nonessential surgery. We reduced our volume from close to 130 operations a day to less than 15.

We’ve made several other, equally significant decisions; probably the biggest was to start accepting COVID-19 patients and provide ventilatory ICU support. We’ve increased our critical care capacity from five to 30 ICU beds, and we have increased our ability to take care of all essential orthopedics throughout the city. We’ve opened an orthopedic trauma triage center in the lobby of the main hospital where the hand and foot center sits. We’ve opened part-time urgent care centers across our satellite locations, which have started to see urgent orthopedic injuries, and we have changed the ninth floor to a negative pressure location to increase safety for the staff who take care of COVID-19 patients.

But we are still unable to increase the capacity of the institution to our goal of 235 patients. In my mind, any empty bed is the equivalent of one person who’s died because we were not able to help.

Most unexpected to me is that in the USA we cannot find masks for people, and we are still arguing about what type of personal protective equipment (PPE) we can provide our frontline workers. It’s like going into the operating room and being told, “They ran out of masks and gloves and you have to just wash your hands a little bit more.”

The other day, somebody said to me that at some point in the future, the COVID-19 crisis as we are experiencing it will just be a story. And the story will be based purely upon the decisions and actions we take right now. We want to make sure that the story is something we’d be proud of. If we can think about the decisions we are making now based upon what it will look like 10 years from now, when people are reading about what we did, we want to make sure that it’s the right story.

### Jennifer O’Neill, DNP, APN, NEA-BC, HSS chief nursing officer; served as chief operating officer overseeing transfer of COVID-19 patients to HSS

Today we are preparing for our first COVID-19 patients, who will arrive later in the week. We’re setting up our ninth-floor operating rooms as ICU rooms. We’re training the staff. We did a critical care simulation of how to transport the patient and provided instruction on issues such as appropriate PPE, how to don and doff, and determining protocols.

For two patients, we’ll have a certified registered nurse anesthetist, two nurses, and either a surgical tech or unit assistant. As we progress, and the census increases, that may increase to a one-to-four ratio. We’re doing “just-in-time training” because many of the patients we are caring for look different from those we had a month ago. Once we bring that first COVID-19 patient over and the team sees we can manage the patient, hopefully there will be a reduction in their anxiety.

Maybe 3 or 4 years ago, at my previous organization, we had a “rule-out-Ebola” patient. It was a Friday night and I was at a Broadway show when the call came in. It turned out that the patient was diagnosed with Lassa fever—an acute viral hemorrhagic illness. Of course, it’s different because that was one patient and now there are thousands, but getting through that allowed me to navigate this situation.

## April 1, 2020

“Grim toll projected, even with distancing.”

*The New York Times,* April 1, 2020.

### David Mayman, MD, HSS orthopedic hip and knee surgeon, served as the chief medical officer overseeing the transfer of COVID-19 patients to HSS

I woke up this morning and thought maybe this is all April Fools’. But it’s not. Today is a big day. When this process began, we were going to help our partner next door, NewYork-Presbyterian (NYP), by taking non-COVID-19 med–surg patients so that they had better capacity to take care of COVID-19 patients. But as this continues to get worse, we knew we needed to do more. We’ve been preparing, but today is day 1. Our ninth floor ORs have been converted into an ICU. Our last walkthrough is at 12:30.

If we get the thumbs up from all the people involved, we will bring our first intubated, ventilated COVID-19 patient to HSS this afternoon.

There’s more anxiety today than we have seen, recognizing that somebody in the hospital is going to be COVID-19 positive, that the chances of us remaining COVID-19 negative are slim. Everybody’s going to be working outside their comfort zone. Today, priority number one is making sure that the staff in the ICU feel ready to take that first patient.

I think back to medical school and residency. One of my mentors said, “David, don’t start with the compromise. Make the right *medical* choice. Everything else, we’ll figure it out.” And to this day, the first thing I think is, “What’s the right thing to do?” Do not start with “Well, I guess this is good enough.” We do that every day with PPE. Because we have to take available supplies into account, there has to be a risk assessment. Yet we always start with “What is the *right* answer?”

A week from now we are going to be right in the thick of it. I think we’ll have some bright news, that daily new cases are going down, which would be fantastic. But hospitalizations will still be going up, intubated- and ventilated-patient numbers will be going up, deaths will be going up. A week from now is going to be rough.

## April 3, 2020

“CDC recommends masks, U.S. deaths rise by more than 1,000 in one day.”

*NBC News*, April 3, 2020.

### Douglas Padgett, MD, HSS associate surgeon-in-chief and deputy medical director

One of the biggest challenges I’ve ever experienced is turning HSS, the biggest musculoskeletal hospital in the world, into a med–surg hospital to help take care of our New York City community. We’re going from an almost exclusively musculoskeletal hospital staffing model to one in which our staff care for medically infirm patients, some with surgical problems as well. Our orthopedic surgeons and musculoskeletal specialists are working in an entirely new environment. There is no playbook, no other example of an orthopedic hospital that suddenly becomes a med–surg hospital. I am nervous but excited.

## April 10, 2020

“Health authorities roll out new tests to gauge spread of COVID-19.”

*The Wall Street Journal*, April 10, 2020.

***Kelly*:** Anticipation is harder than reality. There’s still anxiety here, but people are just going to work now, just acting like doctors and nurses. If you see anyone with a fearful look, you can assume it’s their first day. They still have the anticipatory fear. And you can confidently tell them, “You’re going to be all right.”

One 82-year-old woman we transferred from NYP had been admitted for COVID-19 at the same time as her husband. Unfortunately, he died just 2 hours before I met her. And she had no idea. The nurses had to tell her, but they did it in such a civilized way—in the way her children wanted—with everyone connected through iPads or baby monitors. It was obviously very sad, but the message was passed on in such a caring way. It was a testament to our nursing staff.

Last night, I spoke with the night fellows. “I know this is not what anybody signed up for,” I told them. “But this is a once in a lifetime opportunity. I encourage you to dive in. You can either be a part of it and benefit from the strength that comes from being in the middle of a crisis, or you can avoid it and worry about what you’re missing out on. Either of those reactions will change you for the rest of your life.”

***O’Neill***: We now have a 34-bed ICU. This includes eight ORs with two patients per room and the rest in our PACU. We set up central monitoring for telemetry and vital signs in each of those areas. We are using cameras, baby monitors, Vocera, and the call bell system.

We’ve successfully extubated four patients. One went to a medical floor and two others will transfer today. One will probably transfer tomorrow. We had proned two patients**—**medically they were worsening. Today we will place tracheostomies in patients. They will still require ventilation but will not require the same amount of sedation as when intubated.

This morning I saw the first patient we extubated—he was waving and smiling. He said he wants to go home. He was intubated for almost 3 weeks. That gives me chills.

For the city, the “curve has flattened,” the number of admissions has decreased—but the mortality has increased. The vent census continues to grow, at a slower pace but it’s still increasing. It’s going to be a while before patients get better.

***Padgett***: The transformation has been miraculous. A week ago I was worrying about how we would transition. But everyone has stepped up—even the security guards at the front gate now not only check IDs, but they distribute masks to staff and patients. Our goal is to keep everyone safe, provide them with what they need, so they can go and do the job that needs to be done with confidence. I’m proud of all that people here have been able to do. “Pivot” is probably the best expression—and they have done this with grace, acceptance, and perseverance. They are on the front lines and the level of engagement has been fantastic.

Everyone’s excited about the fact we have made it this far, but it’s not over yet. When we get to the other side, we’ll look back and say, What did we learn from this? Technology, telehealth, and telemedicine have totally changed how we do things. Six weeks ago, I had almost never heard of coronavirus. And now what we have learned is the basis of planning for the next pandemic, whatever it may be.

***Mayman***: I see the data right now as promising. A lot of my friends, not in the medical community, hear that the number of deaths is as high as it’s ever been and they are asking, How can you say we are doing well? I try to explain that although our numbers of new cases and hospitalizations are dropping, the number of people being ventilated is still slowly going up and we are seeing people dying. Those people got this 2 or 3 weeks ago and are now concluding their course with this disease. That die was cast weeks ago.

The highs and lows are exact opposites. We’ve had two ICU patients extubated in the last 24 hours. We’ve had some people from our COVID-19 unit on the eighth floor discharged home. That’s the high. We’ve also had people die. That’s the low.

I’m Canadian, I moved to New York City for my fellowship and stayed. There’s a sense that it’s a huge population but a small community. Unless you live here, it’s difficult to understand. But every New Yorker knows this feeling. This whole idea of “one city” right now really resonates.

I have the privilege of being able to sit in on the COVID-response meetings every day. And every day [HSS president and CEO] Lou Shapiro’s first question is, How is the health of the staff at HSS? That’s first and foremost on his mind. In general, people have held up very well. Everybody’s stressed, and everybody has a moment when they lose it a little bit. But at this point you recognize that it’s okay.

Right now the work is practical. It’s real. We’re addressing questions like: How do we clean an operating room? What’s our air turnover rate? How do we make sure that we have got positive pressure with laminar flow in the operating rooms? It feels really good to say we are tweaking what we have already built, which is different from, Wow, we have got a lot of building to do.

## April 20, 2020

“Single day death toll in New York dips below 500.*”*

*The New York Times,* April 20, 2020.

***Kelly***: I would say that 4 weeks ago, when we made the decision to stop all essential surgery, we were ahead of the curve. We made that decision before almost all of the other hospitals in the New York City area. And at the time the question was always, Are we overreacting? Do we really need to do this much? Lou Shapiro and I agreed that if we look back on this 6 months from now and we are criticized because we overreacted but we had a better outcome than if we’d underreacted, we’d rather be criticized for overacting.

When we started making these transitions, Lou organized livestreams, daily emails, and videos to enable communication with staff as transparently as possible. These have been critical to ensure the staff is part of the decision-making process. They’re very, very appreciative of this communication. My advice to another institution is make sure you are not making decisions in a vacuum; people want to know the reasoning behind decisions.

This is an opportunity for HSS to do things that otherwise may have taken years to accomplish. On March 15, the day before we started instigating a lot of these changes, we had zero telemedicine visits being done daily. And today we are up to up to around 500, which is going to transform the way we interact with patients. Many of the changes we have made may continue going forward because they enable patients to access our healthcare system. Not only will we get through this, but HSS will be a stronger, more united organization as a result.

## April 30, 2020

“As New York morgues ran out of space, a funeral home filled U-Haul trucks with dozens of bodies, police say.”

*Washington Post*, April 30, 2020.

***O’Neill:*** When I was a new grad back in the nineties, I had to take care of my grandfather as he was dying. No sooner had my family told the doctor that that we needed to let him go, that it was okay to take him off all the medication, than my grandfather took his last breath. That memory is in my mind every day. Patients hear everything. They may not be able to communicate, but they still hear, feel, and listen. That moment made me a more compassionate person and nurse. I personally experienced a family member actively dying and understood how people need to be cared for at the end of life. That’s what we have done here at HSS. That’s what I’m most proud of.

## May 1, 2020

“New York City, once the U.S. epicenter of coronavirus, eyes June reopening.”

*The Washington Post,* May 1, 2020.

***O’Neill****:* The most profound experience I’ve had in 6 weeks has been watching how caring and compassionate our nurses are. They do not want anyone to die alone. They make sure that there’s always a provider in the room, holding a hand, talking to the patient. They FaceTime with families who want to share their loved one’s last breath; they make themselves available.

We’ve come together. I can see in the nursing staff that their confidence has increased. There has definitely been an advancement in their scope of practice. Some of the staff want to know if they can remain step down when COVID-19 goes away. They’ve said, “Look, we don’t need to send any patients to NYP. We can manage them here!”

## May 5, 2020

“More than 71,000 people have died from coronavirus in the US.”

*CNN*, May 5, 2020.

**Fig. 2:**
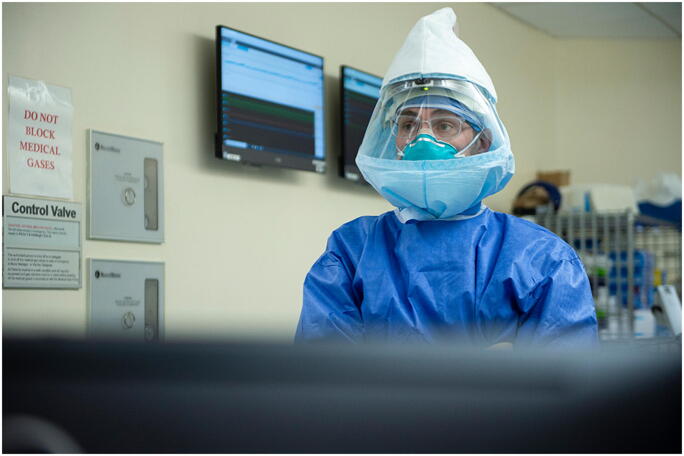
Stavros Memtsoudis, MD, PhD, HSS anesthesiologist: “Obviously, we will never treat each other the same way again. It may sound cheesy, but across the world, it’s like everyone’s in the same boat. We feel for and learn from each other.” Photo by Clay Woodland Englehart.

***Mayman***: Solutions, not problems—I tell my kids that all the time. Do not dwell on the *problem*. Instead, ask: How do we fix it? We have some intensive care–trained intensivists, anesthesiologists (Fig. 2), but they have never taken care of COVID-19 patients . A few of them went across the street to the ICU at NYP. They transferred that knowledge back here. Historically, the nurses here take care of patients who are healthy and recovering from surgery. They too went to the ICU to learn. Within hours they said, “Okay, this is our job now. This is what we need to do.”

We talk about what we need to do to feel that we are safe here. We need capacity. We need the ability to test. By quickly pouncing on an area where we start to see cases and tracing those people and shutting down small areas, we’ll minimize the second wave. We’re not running around trying to find PPE anymore. Our testing ability is getting much better and will continue to improve. We’re more prepared than we were 2 months ago.

Eight weeks ago, [patients] were asked if they wanted to do telehealth. The responses were, “Nope, I’m not good with technology” or “I’m too old for that.” That’s all gone away. Now I do Zoom calls with my parents who are in their 80s. Patients are like, I do not have to drive into the city. I do not have to park a car. I do not have to sit in a waiting room for an hour. I can just get an X-ray locally, upload it to your system, and talk to you from home. And it’s just a half hour instead of my whole day. Patients really like it.

I’ve been doing telehealth, and tomorrow’s my first day back seeing patients in the office again. Now the questions are, What PPE do I have to wear? What’s it going to feel like when I go in to see a patient and I’m wearing a mask and eye protection? What does that eye protection look like, big ugly goggles?

We know that in other parts of the world COVID-19 patients having surgery have had very high complication rates, which is why we’ll be doing a lot of testing. We do not know 100% what recovery means yet. How long is recovery? What are you like 3 months after you had it, 6 months after? When a person is antibody positive, we’ll be asking, Do they have clotting issues? Do they have lung issues? Do they have kidney issues? Do they have cardiac issues? We’re going to be following all of that.

## May 20, 2020

“A new entry in the race for a coronavirus vaccine: Hope.”

*The New York Times,* May 20, 2020.

***Kelly***: Transitioning back into a subspecialty orthopedic institution is a much more intricate process than turning into a COVID-19 hospital. It’s like turning the Titanic 180° on a dime and trying to go in the opposite direction.

We’ve put a number of measures in place to make sure we are not placing any of our patients at risk. We’ve created a consolidated model for seeing patients, so we can limit the number of people who are entering and exiting the building. We’ve instituted rigorous testing policies to make sure we are not operating on patients who are actively exposed or COVID-19 positive. [Executive vice president] Lisa Goldstein and her team have done an incredible job floor by floor, making sure everything is completely terminally cleaned and tested. I feel confident that, right now, it’s safer to be here than it is to be out in the community.

COVID-19 is one of the least understood viruses we have ever seen. Early evidence suggests that it could have a tremendous negative impact on patients in general, and there are inflammatory mediators and cytokine storms that affect multiple organ systems. And then you throw surgery into the mix. Can we identify biological markers? Are there genetic predispositions that increase the likelihood of adverse outcomes after exposure? When you are treating people in their 70s and 80s for debilitating arthritis or spine problems leading to neurological deficit, you start to see this conflict between the urgency of the surgery and concerns about adverse outcomes related to comorbidities.

HSS has the highest concentration of orthopedic patients in the world, and we have the infrastructure to understand the immunological and rheumatological components of COVID-19 better than anywhere else. Ultimately, HSS will have the most significant influence on understanding how orthopedic patients respond to surgical procedures in the COVID-19 era.

## Letters from the Front Lines

### ***Island of Misfit Toys***

As a clinical nurse in the post-anesthesia care unit (PACU), my job is to keep the movement from the OR into the recovery room going smoothly. Before COVID-19, we averaged 60 to 85 cases a day. Then the COVID-19 pandemic reached New York City. In a week’s time, we had 16 ventilators and took our first COVID-positive vented patient.

Because of how sick the COVID-19 patients were, our nurses were in the rooms 10 hours out of a 12-hour shift. Every day we got a list of phone calls from the families who wanted to FaceTime or Zoom with patients. Initially it was for them just to see their family members, to hear their voices, but then we were doing last rites with priests. We had two or three iPads going sometimes and family members crying and saying goodbye.

Eight patients died. I went to the morgue six times. I’ve worked at other hospitals and dealt with death. If I could protect somebody from having to deal with that, I’d rather do so. And I had attending surgeons coming with me. We all did what we could to protect each other.

The intensivists gave the patients the best chances they had. What was so special about the ninth floor? We really wanted to be there. We were like the Island of Misfit Toys. The funny part—all you could see was people’s eyes. So you could not recognize anybody’s face, but you had each other’s back. It was bittersweet when we closed. We do not miss COVID-19. We do not miss the sick patients. But we miss the family we became up there.

What’s our new normal? We’re testing every patient for COVID-19 now before surgery. These masks aren’t going anywhere anytime soon. The new normal is washing your hands 9000 times a day, using a hand sanitizer 3000 times a day. Why go backward?

**Fig. 3:**
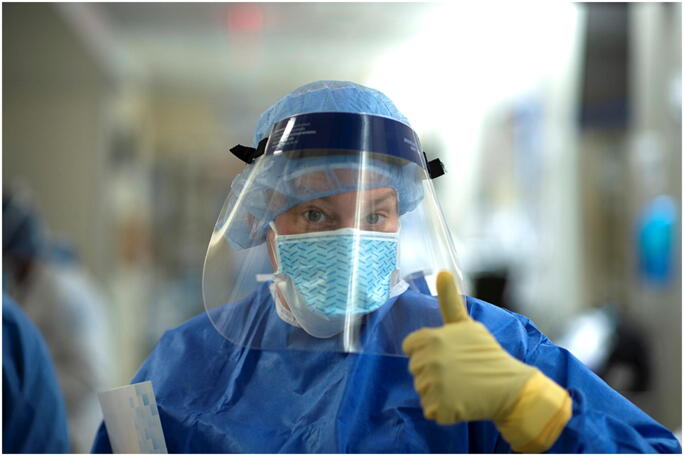
Erin Elder-Niklinski, RN, BSN, CPAN, ONC, post-anesthesia care unit nurse at HSS: “I don’t know what’s going to happen. But what I do know is that we’re prepared.” Photo by Clay Woodland Englehart.

I do not know what’s going to happen. But what I do know is that we are prepared. If we turned our OR into an ICU in a week, I can imagine what we could do now in two days (Fig. 3).—*Erin Elder-Niklinski*, *RN*, *BSN*, *CPAN*, *ONC*

### ***Learning to Swim, Quickly***

I do not even know what today’s date is.

These have been some of the hardest weeks imaginable. People have compared it to a war. But it has been amazing to see how we all pulled together, especially my critical care colleagues. There was no hesitation. I do not like the word “hero,” but this is probably the one point in our lifetimes in which we can say that our profession is the most important one out there. That’s a lot of pressure, but there’s a lot of pride behind it, too.

As HSS was preparing the facilities for our new role, a number of us [anesthesiologists] decided that while some were knee deep in preparations it would be useful for us to help our colleagues at NYP and learn what was to come. So Elaine Yang, Meghan Kirksey, and I went to work in the newly opened operating room ICUs. It felt like we were thrown into the water and had to learn how to swim very quickly. There was no inservice or time to get to know the environment. On my first day, I was the ICU attending caring for over 20 patients and my team consisted of dental hygienists, urologists, pain fellows—people you would not normally see in that environment. We were using anesthesia machines as ventilators. There was a lot of emotional stress but we supported each other and were texting at 2 AM because we knew that nobody could sleep.

All of us anesthesiologists had trained in regular ICUs, trauma ICUs, so we were familiar with all the concepts. But this was different and we had not practiced like this at HSS before: procedures like tracheostomies; taking care of the sickest patients for weeks at a time; nurses using medications that they have not used in so long or ever; talking to families who could not be there themselves every day, keeping them abreast and giving them comfort.

The anesthesiologist’s role has always been one of leadership and involved taking everything into consideration in the operating room. If we need a nurse to do something or the team needs help from the outside—like blood products, equipment, specialists and so on—we’ll be the ones who make those calls. Likewise, we were the ones who planned patient care during the crisis. Our scope has expanded because of this complex patient population. Over the past few weeks, we have been leading teams of up to 20 people during rounds and throughout the day.

Obviously, we will never treat each other the same way again. It may sound cheesy, but across the world, it’s like everyone’s in the same boat. We feel for and learn from each other. There are countries that we pooh-poohed before that have done an amazing job. Perhaps we can learn from them and embrace new ideas and understand that there may be a better way to do things.—*Stavros Memtsoudis*, *MD*, *PhD*, *is the director of critical care services and an anesthesiologist and critical care physician at HSS.*

### ***Out of Bad Comes Good***

For many years, I’ve said, “Out of bad comes good.” That’s what happened back in World War II. The casualties of that war brought on a new life in medicine, particularly orthopedics. For example, some of the German prisoners held by the United States had femur fractures that had been fixed with “Künstler rods,” long metal rods that German doctors placed in the femur itself. This was the beginning of internal fixation for fractures of long bones. American doctors learned from this and, since then, many different rods have been developed.

Also amputees: there were thousands of amputees who were treated in military hospitals, particularly Walter Reed Army Medical Hospital. T. Campbell Thompson, HSS’s surgeon in chief after the war, was the head of the amputee clinics in the military, and that became a very important part of his orthopedic research and training—and also his clinical experience in his active years.

That brings us to the boom in total joint surgery that occurred some years later. In England, John Charnley, a British orthopedic surgeon and regimental medical officer in WWII, developed a “total hip,” which replaced both sides of the hip, the femoral head and the acetabulum. Unlike earlier procedures, the total hip significantly reduced rehab time and allowed people to get up and walk immediately. Philip D. Wilson Jr. visited Charnley and brought total hip replacement surgery to HSS in 1967.

However, when we started doing total hip replacement, there was a significant infection rate. It was difficult to treat patients with infected implants—in fact it wasn’t unusual for a patient who’d had a total hip to be hospitalized for about three weeks. Consequently, infection became an important part of the culture of HSS, and remains so today as infections become more challenging.

These breakthroughs all came about after World War II. So: good out of bad. The culture of medicine has changed because the world is changing and, right now, the world as we know is in a major catastrophe. But something good will come out of it—if nothing other than a realization of the importance of science for our future.—*David B. Levine*, *MD*, *an orthopedic surgeon from 1966 to 1995 and the former chief of scoliosis at HSS, authored* “*Anatomy of Hospital: Hospital for Special Surgery 1863–2013*,” *the definitive history of HSS.*

### ***Sitting with Fear and Love***

It was as if we woke up on March 15 and suddenly there was a new way of doing everything. I was at HSS during 9/11 and that was a very different time. Back then, the staff stood ready, but there was no one to care for; those we waited for had already died in the towers. During COVID-19 we did not have a moment to stand.

For a minute, it felt out of control. No two patients were alike. They came in with different religions, languages, and cultures. We had Catholic patients who did not want to take their rosary beads off when they were being intubated. We had young adults making care decisions for parents, although they were totally unprepared to do so. We had teleconferencing calls with the loved ones of patients as they were dying. It was humbling. Unbelievable.

We developed a family management team that assigned each one of us to talk with a patient’s family member and do what we could to help. “Could you please sit with my mother?,” they would ask. “Could you please tell my father I love him? Could you please just hold his hand?”

And there was Theresa. She had survived breast cancer. She had a very positive attitude. She relied on hope and her own experiences, knowing she had already made it through something very difficult. Her husband could not believe that, after all she had gone through, she now had COVID. He saw that Theresa’s great strength and hope. “You seem to be the same way,” I said to him. “I am,” he said, “but it’s because of her.” He had begun to question God. We had long talks about this.

Theresa did not deny the possibility of dying. She simply said, “I’m just not going there right now.” She talked about it like one would talk about going to the movies. Her faith was so strong. God was there with her the whole time—and she got well! I felt so blessed and honored to be part of her journey.—*Sister Margaret Oettinger*, *OP*, *is the director of spiritual care at HSS.*

### ***Calm Within the Storm***

Before COVID-19, HSS staff were very comfortable taking care of healthy patients who did well after surgery. It was a very pleasant, not very difficult life and I think everybody here was used to it. That changed instantaneously when we stopped doing surgery and instead decided to accept patients from other hospitals where there was a lot of overflow.

This change was traumatic because we were not accepting healthy patients. At first, we accepted patients who were COVID-19 negative but with multiple comorbidities. And then we began accepting patients who tested positive for COVID-19. This caused tremendous stress for the staff.

Dealing with these patients required changes to our processes. Initially, each patient’s code status defaulted to full code—after all, we did not want anyone to die. Halfway through we changed that to code status “not on file” and encouraged staff to ask patients if they wanted to be a full code or do not resuscitate. These discussions were difficult for our staff, who were not used to asking, do you want to be resuscitated? Do you want artificial means of feeding?

But the staff did get very good at assessing which patients required more oxygen. Our goal was to avoid a code at all costs because the chance for mistakes in a code situation is so much greater. We tried to have anesthesia intervene as early as possible so patients could be intubated in a calm environment.

My mentor, Charles Christian, was chair of rheumatology and a physician in chief at HSS. He would always say, If you put your shoes on and walk down the hallway in a positive manner, people will usually follow. In fact, I had this exact experience during the crisis. A couple of weeks after we began admitting new patients, I walked past an office where the doctors were eating lunch and the mood was upbeat—there was even some laughing going on! I knew that day that it was going to be okay.—*Linda Russell*, *MD*, *is the director of perioperative medicine at HSS.*

### ***Toward a New Normal***

Back in March, we identified what needed to happen to convert this institution from an orthopedic hospital into a general med–surg hospital. And pretty quickly, we turned off the entire enterprise, moving from hundreds of orthopedic procedures [a day] down to just a few. Frankly, turning it off was easy. Turning it back on has been incredibly complicated.

As we move toward a new normal and reengage with patients who need musculoskeletal care, our working group has been focusing on the two most important issues: establishing testing and safety protocols. But we are not sure what the landscape is right now, so there’s trepidation—is this the right time to go back? Over decades of spine, joint replacement, and sports medicine surgeries, our patients have been, for the most part, healthy. We know how to screen and monitor them. But prior coronavirus exposure may increase risk for complications after surgery. Will there be things that prolong their recovery? This is a great unknown. We’ll need to maintain vigilant awareness of possible adverse outcomes.

We’re slowly getting back into doing urgent surgery; we are defining this as orthopedic surgery that’s been deferred through the pandemic, for conditions that have affected the patient’s ability to do activities of daily living. Closely monitoring outcomes will be more important than ever.

We know that comorbidities can affect how a patient recovers after surgery. So in this first phase of getting back to our new normal, we are looking at doing urgent procedures in patients meeting certain criteria. And we have done a good job at risk stratifying, making sure that the patients we are taking care of right now have the lowest potential risks for complications.

Over the past few months, I’ve gained a deeper appreciation for what it takes to run this institution. Before the pandemic, as a surgeon, I took operations for granted. I knew that engineering and environmental services kept the place clean, that finance kept the place running, but I never considered the full scope of their responsibilities. This pandemic made me realize the importance of working together.—*Douglas Padgett*, *MD*, *HSS associate surgeon-in-chief and deputy medical director.*

### ***Advancing Research***

We knew the epidemic was coming, but not how quickly it would change everything. The first few weeks of executing administrative changes were intense. But, I’m a researcher at heart. So, I was—at the same time—reading all the data and the descriptions of COVID-19 that were so rapidly being put into the public domain. The pace of research was remarkable. It was quickly apparent that some of the mechanisms of COVID-19, particularly in patients who developed severe disease, were based in the very same kinds of immunology mechanisms that we see in lupus and other systemic autoimmune diseases.

My own research over the last 20 years has been focused on interferon—the key host defense mechanism that the body puts out in the context of a virus infection. What role was interferon playing in COVID-19? How did the immune system shift from a protective response to one that was inflammatory? Review of the data and knowledge of disease mechanisms gave us some ideas about therapeutic approaches, and we launched a multidisciplinary committee at HSS to identify therapeutic targets and consider developing therapeutic trials across a spectrum of immune system mechanisms. Some of these targets developed into internal protocols for our immunologists and our infectious disease colleagues. Some we developed in collaboration with biotech and pharmaceutical companies. Some are going forward at NYP.

I’ve also been working closely with one of our junior rheumatology colleagues to create a preliminary case series on the administration of an anti-inflammatory medication to COVID-19 patients at NYP. This case series, describing the use of anakinra (recombinant IL-1 receptor antagonist) in COVID-19 patients with evidence of cytokine storm and respiratory failure, was published in *Arthritis & Rheumatology*. Iris Navarro-Millan, a rheumatologist in our division, is developing a controlled trial of this drug that we hope to take forward in COVID-19 patients at NYP.

The ability to study the blood of COVID-19 patients is a great opportunity to learn in detail how the immune system is working. We can then compare our observations to the data we have already collected on, for example, our lupus patients. What’s the same and what’s different? Research like this may have significant consequences for patients with this frightening and life-threatening disease.—*Mary* (*Peggy*) *K*. *Crow*, *MD*, *outgoing HSS physician-in-chief and chair of the Department of Medicine*.

